# Acute pancreatitis as a complication of routine colonoscopy—A rare case report

**DOI:** 10.1016/j.ijscr.2019.03.007

**Published:** 2019-03-19

**Authors:** M Masood Sidiqi, Bill Gong

**Affiliations:** General Surgery, Rockingham General Hospital, Western Australia, Australia

**Keywords:** Pancreatitis, Colonoscopy, Endoscopy, Colonoscopy-induced, Complication

## Abstract

**Introduction:**

Abdominal pain after colonoscopy is a relatively common symptom and usually benign. Colonoscopy-induced pancreatitis is an extremely rare phenomenon that can sometimes be missed leading to delayed diagnosis and treatment.

**Presentation of case:**

A 53 year old woman presented to the Emergency Department with abdominal pain, a significantly raised lipase and a CT scan revealing pancreatitis. She had no previous history of pancreatitis or any aetiological risk factors. Her pain started 2 h after having a routine outpatient colonoscopy for polyp surveillance. The endoscopist had no difficulty during the procedure and the findings were unremarkable. She developed a Systemic Inflammatory Response Syndrome (SIRS) and an ileus requiring a prolonged hospital admission. However with conservative management she improved and was discharged on day 11 post-admission in stable condition.

**Discussion:**

The mechanism of colonoscopy-induced pancreatitis is not well understood. Hypotheses include mechanical trauma to the pancreas caused by the endoscope particularly at the splenic flexure, over-insufflation of the colon, external abdominal pressure, and transmural colonic burns via electrocautery causing irritation to the pancreas.

**Conclusion:**

Pancreatitis should be considered in the differential diagnosis of abdominal pain post-colonoscopy after the more common explanations are excluded.

## Highlights

• Pancreatitis should be considered a rare potential complication for patients who develop acute abdominal pain after colonoscopy.
• Procedural difficulties particularly around the splenic flexure, transmural colonic burns, and over-insufflation of the colon may increase the risk of pancreatitis.
• This rare complication should be considered in discussions when obtaining informed consent for the procedure.

## 1 Introduction

Colonoscopy is a very common diagnostic and therapeutic procedure for investigation of colonic pathology. Well recognised complications include perforation, bleeding, post-polypectomy syndrome and side effects related to sedation and analgesia. However there are also a number of rare complications reported in the literature including splenic trauma, infection, diverticulitis and appendicitis. Pancreatitis is a well-documented complication of endoscopic retrograde cholangiopancreatography [1], but generally not associated with endoscopic procedures that do not involve ampullary cannulation, far less so colonoscopy. To the best of our knowledge, there have only been four reports in the English language literature of colonoscopy-induced pancreatitis [[2], [3], [4], [5]]. Most of these cases were on patients who either had a previous history of pancreatitis, significant risk factors, or a technically difficult colonoscopy. We report a case of severe pancreatitis on a fit and healthy patient, after a routine and straightforward colonoscopy. The work has been reported in line with the SCARE criteria [6].

## 2 Presentation of case

A 53-year-old otherwise fit Caucasian woman underwent a routine colonoscopy for polyp surveillance. Her past medical history revealed gastro-oesophageal reflux, dyslipidaemia, hip bursitis, and laparoscopic cholecystectomy two years ago for cholelithiasis. She was a non-smoker, rarely consumed alcohol, and had no drug allergies. Her regular medications were esomeprazole and atorvastatin which she had commenced 3 years ago. The procedure itself was not technically difficult and deep abdominal palpation was not performed during the colonoscopy. A scar was seen at the site of a previous polypectomy in the transverse colon with no residual polyp. A 2 mm ascending colon polyp was removed with a cold snare and no electrocautery was used throughout the procedure. She was premedicated with midazolam, propofol and alfentanil.

**Fig. 1:**
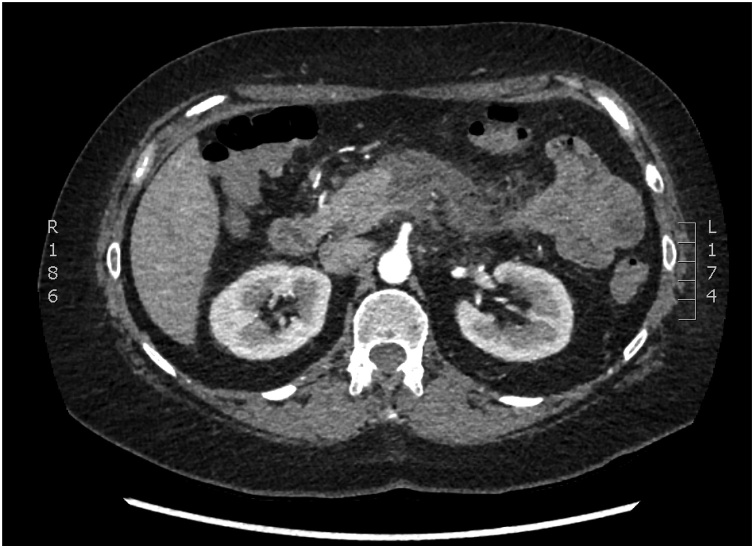
CT Abdomen revealing marked inflammatory stranding around the body of the pancreas, consistent with pancreatitis.

The patient developed an acute onset of abdominal pain 2 h post procedure associated with generalised cramping. She was observed in the endoscopy unit for a few hours before being discharged. The pain persisted and she presented to the Emergency Department of our institution the next day after developing nausea and vomiting. She had minimal flatus and could not pass any bowel motions. On physical examination all her vital signs were normal and her abdomen was tender in the epigastrium without any signs of peritonitis. Initial laboratory investigations revealed a white cell count of 13.65 × 10^9^/L (normal 4–11 × 10^9^/L), C reactive protein of 67 mg/L (normal <5 mg/L), and a lipase of 809 U/L (normal 20–210 U/L). Haemoglobin, liver function tests, calcium and lipid profile were all normal. Computed tomography scan of the abdomen showed inflammation in the body of the pancreas, with peripancreatic stranding, and a small amount of surrounding fluid consistent with acute pancreatitis (Fig. 1).

No CBD dilatation or stones were identified, nor any signs of pneumoperitoneum. The pancreas did not show any structural anatomical anomaly.

Management consisted of complete bowel rest, intravenous fluids, antiemetics, analgesia and prophylactic antibiotics. She developed fevers and ileus during her prolonged admission, and her CRP rose up to 270 quantifying a severity score of “severe pancreatitis” according to recent studies [7,8]. She subsequently had a repeat CT abdomen which did not show any signs of pancreatic necrosis, pseudocyst, pseudoaneurysm, or fluid collection. As she showed clinical and biochemical improvement over the next week, her diet was escalated back to normal and she was discharged day 11 post presentation in stable condition.

## 3 Discussion

Low grade pancreatic inflammation post endoscopy or colonoscopy may be more common than previously reported. Prior studies reported asymptomatic hyperamylasuria occurring in 6.6% of patients undergoing endoscopy, while hyperamylasaemia was reported in 12%. However this was thought to be secondary to increased secretion of the salivary isoenzyme of amylase, and none of the patients in the studies developed clinical pancreatitis [[9], [10], [11]]. A literature review reveals only four published case reports of acute pancreatitis post-colonoscopy [[2], [3], [4], [5]]. Two of these cases report a technically challenging procedure where the endoscopist found difficulty passing the splenic flexure and multiple attempts to insert the colonoscope were made [2,3]. While a subsequent case did not report this difficulty, CT imaging demonstrated haemorrhage around the tail of the pancreas, and given its proximity to the splenic flexure, mechanical trauma was thought to be the cause [4]. The most recent case report demonstrated pancreatitis post-colonoscopy on a patient with multiple risk factors, including a previous history of pancreatitis, inflammatory bowel disease, and immunosuppressants, all of which suggest a possibility of multifactorial aetiology to the disease [5].

Although the underlying mechanism of pancreatitis in such cases is unclear, the proposed hypothesis is mechanical trauma to the tail and body of the pancreas caused by movement of the endoscope. In addition, excessive bowel distension due to gas insufflation may cause pressure to the pancreas. Similarly, external pressure on the abdomen may also provoke local trauma and an inflammatory response. Another possible explanation is that electrocautery during polypectomy can cause transmural colonic burns which may result in pancreatic irritation, as suggested in a previous report [9].

In the case we have reported, our patient was fit and healthy and had a routine colonoscopy. The procedure was technically straightforward with easy passage through the splenic flexure, without external abdominal pressure or the use of electrocautery. She did not exhibit any of the usual aetiological risk factors associated with pancreatitis. She had a previous cholecystectomy in 2016 (with absence of ductal stones on imaging) and had not consumed any alcohol for at least 6 months before her colonoscopy. She was tested for autoimmune pancreatitis, lipids and a metabolic/electrolyte workup, all of which were negative. Her bowel preparation was tolerated well with no symptoms to suggest dehydration contributed to her developing pancreatitis. Her imaging showed no anatomical anomalies of the pancreas, and she denied any abdominal trauma prior to her procedure.

It is highly unlikely that the atorvastatin was the cause of her pancreatitis, as the patient had been on treatment for more than two years. As rare as statin-induced pancreatitis is, most published case studies report that the onset of symptoms usually occur within months after commencement of therapy [12]. Furthermore, the patient was rechallenged with atorvastatin during admission and her symptoms did not deteriorate.

## 4 Conclusion

Abdominal pain after colonoscopy is a relatively common symptom and usually benign. Pancreatitis should be considered in the differential diagnosis after the more common explanations are excluded. Awareness of this complication can help initiate early diagnosis and treatment.

## Conflict of interest

Authors have no conflict of interest to disclose.

## Funding

None.

## Ethical approval

Ethical approval is not applicable.

## Consent

Written consent was obtained from the patient for publication of this case report and accompanying images. A copy of the written consent is available for review by the Editor-in-Chief of this journal on request.

## Author contribution

Dr Masood Sidiqi contributed in medical record review, literature search, and writing of the draft. Dr Bill Gong contributed towards review of the paper.

## Registration of research studies

None.

## Guarantor

Both authors have read and approved the manuscript and accept full responsibility for the work.

## Provenance and peer review

Not commissioned externally peer reviewed.
